# Age and sex distribution of *Mycobacterium tuberculosis* infection and rifampicin resistance in Myanmar as detected by Xpert MTB/RIF

**DOI:** 10.1186/s12879-021-06296-0

**Published:** 2021-08-09

**Authors:** Marva Seifert, Hlaing Thazin Aung, Nicole Besler, Victoria Harris, Tin Tin Mar, Rebecca E. Colman, Timothy C. Rodwell, Si Thu Aung

**Affiliations:** 1 University of California San Diego, 9500 Gilman Dr, La Jolla, CA 92093 USA; 2 Clinton Health Access Initiative, Yangon, Myanmar; 3 FIND, the global alliance for diagnostics, Campus Biotech, 9 Chemin des Mines –, 1202 Geneva, Switzerland; 4 Ministry of Health and Sports, Office No. 4, Naypyitaw, Myanmar

**Keywords:** Myanmar, *Mycobacterium tuberculosis*, Rifampicin resistance, Xpert MTB/RIF, Sex, Age

## Abstract

**Background:**

Detection of tuberculosis disease (TB) and timely identification of *Mycobacterium tuberculosis* (*Mtb*) strains that are resistant to treatment are key to halting tuberculosis transmission, improving treatment outcomes, and reducing mortality.

**Methods:**

We used 332,657 Xpert MTB/RIF assay results, captured as part of the Myanmar Data Utilization Project, to characterize *Mtb* test positivity and rifampicin resistance by both age and sex, and to evaluate risk factors associated with rifampicin resistance.

**Results:**

Overall, 70% of individuals diagnosed with TB were males. Test positivity was higher among males (47%) compared to females (39%). The highest positivity by age occurred among individuals aged 16–20, with test positivity for females (65%) higher than for males (57%). Although a greater absolute number of males were rifampicin resistant, a greater proportion of females (11.4%) were rifampicin resistant as compared to males (9.3%). In the multivariate model, history of previous treatment, age less than 30, testing in the Yangon region, and female sex were significantly positively associated with rifampicin resistance after adjusting for HIV status and year test was performed.

**Conclusions:**

Our results indicate that young adults were more likely to test positive for TB and be identified as rifampicin resistant compared to older adults.

## Background

Tuberculosis (TB) disease remains the leading cause of death from a single infectious agent globally, and although the number of underreported and underdiagnosed cases continues to trend downward, the gap between incident cases and reported cases in 2019 was estimated to be approximately three million worldwide [1]. This gap in disease detection not only contributes to ongoing transmission, but also reduces the probability of treatment success, particularly for individuals infected with strains of *Mycobacterium tuberculosis* (*Mtb*) that are drug resistant. In 2018 the World Health Organization (WHO) estimated that only one in three individuals resistant to rifampicin and isoniazid, known as multi-drug resistant TB (MDR-TB), were enrolled in appropriate treatment [1, 2], due to the lack of accurate and timely diagnostic solutions for drug resistance detection. The recent introduction of rapid molecular based methods that simultaneously detect the presence of *Mtb* and rifampicin resistance has provided an opportunity to reduce this diagnostic gap. Understanding factors associated with missed cases and identifying sub-populations at increased risk for drug resistance will allow for more efficient utilization of limited programmatic resources.

Globally, TB incidence is significantly higher in males compared to females, and in 2018, the WHO reported that that 56% of the global TB burden was among adult males, 32% among adult females, and 12% among children [1, 3]. Although country-specific variation in sex distribution of incident TB has been identified [4], incomplete age disaggregation of TB surveillance data has complicated disease burden estimates for young adults and has hampered our understanding of risk of both TB disease and drug resistance among these age groups [5]. Additionally, previous treatment for TB has emerged as the most significant predictor of MDR-TB worldwide, however a growing proportion of individuals without a history of previous treatment are being diagnosed with MDR-TB, indicating the need for more resistance testing in groups not traditionally at risk of drug resistance [6–9]. There is less consensus regarding other risk factors associated with resistant disease, and while multiple studies indicate that female sex [7, 8, 10] and younger age [6, 7, 11] are associated with increased risk of MDR-TB, other studies demonstrate that older age [12] and male sex [13, 14] are associated with increased risk of resistance. This lack of granular country-level data of both age and sex distribution of disease continues to hamper programmatic targeting for both *Mtb* case finding and resistance detection.

While the WHO has ranked Myanmar in the top 14 countries impacted by a trifecta of increased risk: high burden of TB disease, HIV/TB coinfection, and multi-drug resistant TB; national TB prevalence surveys in Myanmar have shown significant reductions in TB incidence over the last decade [1]. Recent scale up of in-country rapid testing capacity using a national network of Xpert MTB/RIF (Cepheid, Sunnyvale, CA, USA) linked to the Myanmar Data Utilization Project has further increased testing capacity and diagnosis of TB and MDR-TB. This expansion of national rapid diagnostic capability and connectivity provides a unique opportunity to explore the characteristics of TB disease by both age and sex, and to identify at-risk sub-populations for additional diagnostic targeting [15–18]. The primary aim of this study was to utilize the electronically captured data to uncover demographic risk factors and patterns associated with TB diagnosis and rifampicin resistance specific to Myanmar. A secondary aim was to use Xpert MTB/RIF results to identify regions of the *rpoB* gene most frequently associated with rifampicin resistance circulating in Myanmar. This analysis of country-wide TB diagnostic data is the first study in Myanmar to rely on Xpert MTB/RIF results to characterize the epidemic on a national level and can be used to inform programmatic strategies to continue to identify and effectively close country-specific diagnostic gaps.

## Methods

### Study data

Xpert MTB/RIF data on *Mtb* detection and drug resistance, together with associated demographic data, were captured as part of the Myanmar Data Utilization and Connectivity Project for the period from January 2015 to December 2018 and used for analysis. Demographic data were entered electronically at GeneXpert deployment sites, and Xpert MTB/RIF results were automatically generated and collated centrally using the GxAlert connectivity solution from SystemOne (Northhampton, MA, USA), as part of the national testing effort. While the algorithms for determining which individuals received Xpert testing evolved over the project period as part of the continued rollout of Xpert MTB/RIF testing in Myanmar, initial inclusion criteria in 2015 for testing in all regions except for Yangon, included all individuals with a history of TB treatment, all HIV positive TB cases, and all MDR-TB contacts. Within Yangon, in addition to previously listed inclusion criteria, all smear positive individuals were also tested using Xpert MTB/RIF. In May of 2015 inclusion criteria expanded for all regions to include individuals with positive smears at two months post-treatment initiation, children under 15 who were able to produce sputum, children under 15 at risk of TB meningitis, all smear negative patients with abnormal radiographic findings, individuals at risk of having drug resistant TB, individuals living with HIV, individuals with diabetes, and persons with a history of TB treatment.

Data were stripped of unique patient identifiers prior to analysis and records missing sex data were excluded. Records containing missing age variables, reported as 99 (coded as missing data), or listed ages of 100 or greater were removed. Age was collapsed into five-year increments, with the exception of individuals ages 80 to 98 which were aggregated into a single age category. The final dataset included test records from 87 deployment facilities across Myanmar and included the following variables: test date, Xpert MTB/RIF assay result (including both *Mtb* and rifampicin resistance detection and the corresponding resistance probe(s)), sex, age category, HIV status, and self-reported history of previous treatment.

### Mtb detection and rifampicin resistance determination

The molecular beacon technology used by the Xpert MTB/RIF assay detects mutations on the *rpoB* gene which are associated with phenotypic rifampicin resistance [2, 19]. Five overlapping probes span the 81 base pair resistance determining region (codons 507–533 in the *E.coli* system or 426–452 in the *Mtb* system) of the gene and are designed to hybridize, or bind, to wildtype deoxyribonucleic acid (DNA). When a probe binds to the wildtype DNA target, the beacon undergoes a conformational change and fluoresces. If two or more of the five beacon probes hybridize and fluoresce, the results are classified as positive and indicate the detection of *Mtb* DNA. Mutations within the target sequence interfere with hybridization of the probe and result in partial or total suppression of the fluorescence of a probe. If one or more of the beacon probes fail to fluoresce the result is classified as rifampicin resistant and the non-fluorescing probe or probes are identified as regions containing resistance conferring mutations [20].

### Statistical analysis

We modeled the proportion of patients by both age category and sex, who tested positive for *Mtb* out of all Xpert MTB/RIF assay results recorded each year, to detect temporal patterns in *Mtb* positivity. Differences in the proportion of *Mtb* positive individuals who were identified as rifampicin resistant were also stratified by both sex and age category to identify higher risk individuals. Additionally, prevalence of specific mutation probes used for resistance determination were assessed both by sex and age category. All study variables potentially associated with rifampicin resistance were initially evaluated using univariate analysis to determine association with rifampicin resistance and then were included in a multivariate logistic regression model to determine association with rifampicin resistance in context of other covariates. Odds ratios and 95% confidence intervals were calculated as measures of association, and *P* < 0.05 was considered statistically significant. All analyses were performed using STATA/SE 15.1 (Stata Corp, College Station, TX, USA).

### Ethics

The need for informed consent was waived and the study was approved as conducted by the Ethics Review Committee, Department of Medical Research, Ministry of Health and Sports, the Republic of the Union of Myanmar (Approval number – Ethics/DMR/2018/155). All analyses were performed in accordance with guidelines and regulations for working with de-identified data. In addition, ethics approval was sought from the University of California San Diego Institutional Review Board, La Jolla, CA, USA, however the study was determined to be exempt from review.

## Results

A total of 339,358 Xpert MTB/RIF results were captured between 2015 and 2018 as part of the Myanmar Data Connectivity Project. Five-thousand, eight-hundred and fourteen (5814) records with patient age missing or recorded as greater than 99, and 886 records with missing or unidentified patient sex, were excluded from the dataset, resulting in a total of 332,657 (98%) records used for analysis. Because data were captured as deployment sites were being added to the project, the number of individuals tested each year using Xpert MTB/RIF tripled during the course of the project, increasing from 42,327 in 2015 to 122,724 in 2018. Forty-four percent (44%) of all Xpert MT/RIF results were positive for *Mtb* and only 5% were invalid, had an error, or gave no result (see Table 1). Of the 147,328 *Mtb* positive results, 10% (14,660) were rifampicin resistant. Approximately 18% of all individuals tested had a record of being HIV positive and 36% reported previous treatment for TB.

**Table 1:** Characteristics of the study population stratified by sex

	Sex	
	Males (*n* = 218,963)	Females (*n* = 113,694)
*Mtb* Positive Result (%)	103,008 (47.0)	44,320 (39.0)
Rifampicin Resistant	9604	5056
Invalid/Error/No result (%)	11,268 (5.1)	6010 (5.3)
HIV Status		
Negative (%)	90,292 (41.2)	44,673 (39.3)
Unknown (%)	89,321 (40.8)	49,855 (43.9)
Positive (%)	39,350 (18.0)	19,166 (16.9)
Treatment History		
None (%)	131,861 (60.2)	73,266 (63.6)
Unknown (%)	6182 (2.8)	3357 (3.0)
Yes (%)	80,920 (37.0)	38,071 (33.5)
Region		
Yangon (%)	81,828 (37.4)	45,791 (40.3)
All other regions combined (%)	137,135 (62.6)	67,903 (59.7)
Test Year		
2015	28,008	14,319
2016	45,548	23,456
2017	65,064	33,538
2018	80,343	42,381

**Fig. 1:**
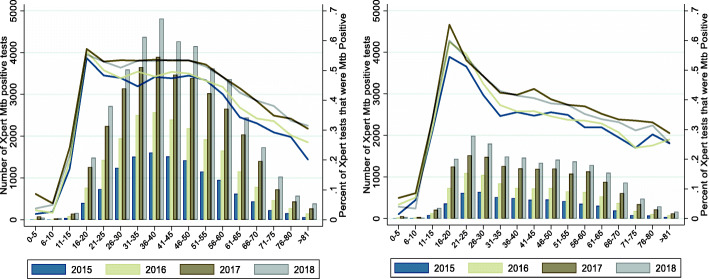
*Mtb* positive results stratified by age category for males (a) and females (b). The yearly counts of *Mtb* positive results for each age category are represented by bars, and the proportion of *Mtb* positive results to total GeneXpert MTB/RIF results for each age category is represented by lines (second y-axis)

A vast majority (70%) of all Xpert MTB/RIF positive individuals were male. *Mtb* test positivity varied significantly by sex; overall 39% of all females tested were *Mtb* positive compared to 47% of all males tested (Pearson chi2 = 147.6, p value < 0.001). Although the total number of assays administered increased each year, the proportion that were *Mtb* positive for each age category remained similar year over year as evidenced in Fig. 1a and Fig. 1b. *Mtb* positive results stratified by year and age category indicate that peak *Mtb* test positivity (57%) for males occurred between ages 16–20 and *Mtb* test positivity remained above 50% until ages 51–55 (Fig. 1a). *Mtb* positivity in females peaked at 65% in the same age range of 16–20, however *Mtb* positivity declined rapidly and fell below 50% by ages 26–30 (Fig. 1b). Median age for receiving a Xpert MTB/RIF test for males was 44 (IQR: 32,55) and for females was 41 (IQR: 27, 55) (data not shown). The male to female Xpert MTB/RIF positive ratio was 2.3.

Rifampicin resistance differed both by sex and age. Overall, among females who were positive for *Mtb*, 11.4% were rifampicin resistant and among males, 9.3% were rifampicin resistant (Pearson chi2 = 147.6288 *P* value < 0.001). Even though the absolute number of rifampicin resistant individuals increased each year during the data collection period, from 2913 in 2015 to 4174 in 2018, the proportion of *Mtb* positive patients identified as being rifampicin resistant declined year over year. For females, detected rifampicin resistance declined from 20.0% in 2015 to 8.3% in 2018, and among males declined from 15.8% in 2015 to 7.1% in 2018.

When stratified by age category, rifampicin resistance was highest among individuals aged 21–25 for both males and females compared to all other age categories. The proportion of rifampicin resistance over the four-year study period among ages 21–25 was 15.7% for females and 13.1% for males. Eighteen percent of previously treated individuals were identified as rifampicin resistant and 7% of individuals with no history of previous treatment were resistant.

Individual probe level rifampicin resistance data was available for 85.1% (12,481) of the reported rifampicin resistant results. Lack of hybridization to one of the five beacon probes (single probe resistance) accounted for 97.2% (12,125) of all resistance calls. The remaining individual probe level resistance was due to lack of hybridization to two or three of the five beacon probes and accounted for 2.4% (305) and 0.4% (51) respectively. There were no significant differences by sex (Pearson chi2 = 1.8, *p* value =0.4) or age category (Person chi2 = 30.8, p value = 0.5) in the total number of individual probes that failed to hybridize for resistance classification.

For single probe resistance, lack of hybridization to probe E accounted for 75.2% (9116) of all resistance calls. This failure of probe E hybridization accounted for 77.9% of all single probe resistance calls among females and 73.7% of single probe resistance calls among males. The most common double probe resistance combinations were probes D and E and probes A and B, which accounted for 32.8% (100) and 25.3% (77) of double probe resistance calls respectively. Lack of hybridization on three probes, A, B, and E accounted for 51.0% (26) of all triple probe resistance calls.

Logistic regression was used to determine if sex, age less than 30, previous treatment, test year, Yangon region, and HIV status were associated with rifampicin resistance (see Table 2). In the adjusted model, female sex, age less than 30, Yangon region, and history of previous treatment were all positively and significantly associated with increased risk of rifampicin resistance. Individuals with a history of previous treatment were 2.85 (95% CI 2.75, 2.96) times more likely than individuals without a history of treatment to be diagnosed with rifampicin resistance, individuals tested within the Yangon region were 1.92 (95% CI 1.85, 1.99) times more likely than individuals from all other regions combined to be diagnosed with rifampicin resistance, individuals age 30 and younger were 1.63 (95%CI 1.57, 1.70) times more likely than individuals older than age 30 to be diagnosed with rifampicin resistance, and females were 1.22 (95%CI 1.17, 1.26) times more likely than males to be diagnosed with rifampicin resistance after taking into account HIV status and year of assay administration.

**Table 2:** Crude and adjusted odds ratios for rifampicin resistance by sex, age, HIV status, history of previous treatment, region, and year of Xpert MTB/RIF assay administration. Total Mtb positive *n* = 147,327

Demographic and clinical characteristics	Rif-Resistant		Crude		Adjusted	
	n	%	Odds ratio	95% CI	Odds ratio	95% CI
Sex						
Male	9604	9.3	reference		reference	
Female	5056	11.4	1.25	1.21, 1.30	1.22	1.17, 1.26
Age						
> 30 years	9881	8.9	reference		reference	
< 31 years	4779	13.1	1.54	1.49, 1.60	1.63	1.57, 1.70
HIV status						
Negative	6720	9.1	reference		reference	
Unknown	6748	11.4	1.30	1.25, 1.34	1.01	0.97, 1.04
Positive	1192	8.6	0.94	0.88, 1.01	0.87	0.82, 0.93
Previous treatment						
None	7766	7.3	reference		reference	
Unknown	375	9.0	1.26	1.13, 1.40	1.31	1.18, 1.47
Yes	6519	17.8	2.76	2.66, 2.86	2.85	2.75, 2.96
Selected Region						
Other regions	6660	7.5	reference		reference	
Yangon	8000	13.7	2.0	1.89, 2.02	1.92	1.85, 1.99
Year of Test						
2015	2913	17.0	reference		reference	
2016	3529	12.2	0.68	0.64, 0.72	0.79	0.75, 0.83
2017	4034	8.8	0.47	0.45, 0.50	0.65	0.62, 0.70
2018	4174	7.5	0.40	0.38, 0.42	0.58	0.55, 0.61

## Discussion

**Fig. 2:**
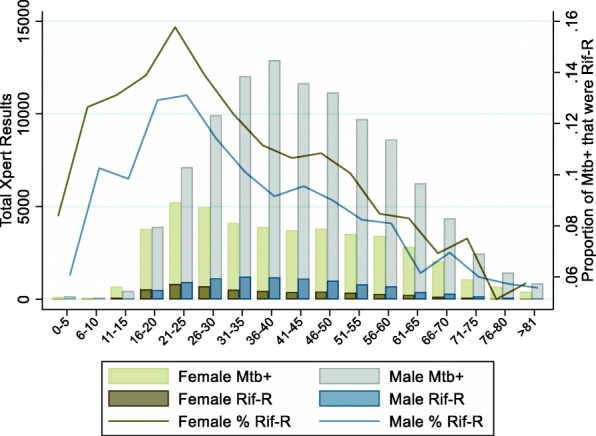
*Mtb* positive results and proportion rifampicin resistant by age category and sex. The total number of Xpert Results by age category are represented by the bars, and the proportion of *Mtb* positive results that were rifampicin positive are represented by lines (second y-axis)

This population-based Xpert MTB/RIF analysis followed expected gender distributions [21]; 70% of individuals diagnosed with *Mtb* were males, and overall, the proportion of males who were *Mtb* positive out of all males tested (47%) was higher than the proportion of females who were positive for *Mtb* (39%), replicating previous smaller scale regional reports from Myanmar [15]. Examination by age category revealed that *Mtb* test positivity differed significantly over life course with the highest proportion of individuals who tested positive for *Mtb* among those aged 16–20 (Fig. 1). Prevalence of rifampicin resistance also differed by age and sex, and although a greater absolute number of males were rifampicin resistant over the period analyzed, the proportion of *Mtb* positive individuals identified as rifampicin resistant overall was greater for females as compared to males, 11.4% versus 9.3% (Fig. 2). Rifampicin resistance was highest among those who had previously been treated for TB compared to those who had no history of treatment (17.8% versus 7.3%); and in the multivariate model in addition to previous treatment, both age less than 30 and female sex were still significantly associated with rifampicin resistance after taking into account HIV status and year test was performed (Table 2). These higher *Mtb* test positivity and resistance rates among young adults provide a potential opportunity to target Xpert MTB/RIF screening among individuals not traditionally classified as high risk [22].

When compared to TB infection models proposed by Seddon et al. and Marais et al., Xpert MTB/RIF results in this population-based cohort exhibited strikingly similar age and sex stratified patterns [23, 24]. Although few children age less than five in our dataset were tested for or diagnosed with pulmonary TB disease when compared to adults, models indicate that this age group is at increased risk of extrapulmonary forms of disease, which would have excluded them from the Myanmar Xpert MTB/RIF testing algorithm. Both absolute numbers of incident cases and *Mtb* test positivity rates were lowest among children aged 5–10 in our dataset, again following proposed models that indicate risk of progression to either pulmonary or extrapulmonary disease falls to its lowest point among this age group. As expected the risk of having pulmonary disease rose dramatically among young adults, likely due to adolescent changes in sex hormone mediated immunological response to infection, increasing their risk of pulmonary disease [25, 26]. In our dataset females exhibited an earlier rise in *Mtb* test positivity as compared to males, resulting in a larger absolute number of *Mtb* cases among females in the 11–15 age group compared to males (Fig. 1). The proportion of individuals testing positive for *Mtb* was highest for both males and females in the age 16–20 group, however *Mtb* positivity remained above 50% for males until age 51–55, in contrast to females where *Mtb* positivity declined dramatically and dropped below 50% by age 26–30, resulting in significantly larger number of males being diagnosed with *Mtb* infection. Although young adults are often considered at ‘low risk’ of TB, our data indicate that additional testing may be warranted in this cohort as their risk of TB and role in transmission may be more substantial than previously understood [27].

Unsurprisingly, over the four-year study period as the number of deployment sites grew and the total number of individuals being tested increased, the total number of individuals identified as being rifampicin resistant year over year increased. However, as the inclusion criteria for Xpert MTB/RIF testing evolved and expanded to include more presumptive *Mtb* positive cases, the proportion of *Mtb* positive patients with rifampicin resistance actually declined for both males and females. Overall, the proportion of rifampicin resistance among *Mtb* positive individuals remained greatest among females compared to males for all age categories (see Fig. 2). The proportion of *Mtb* positive individuals that were identified as rifampicin resistant peaked for both males and females age 21–25, at 13 and 16% respectively, which is consistent with other studies identifying increased risk of rifampicin resistance among young adults [7, 13]. Approximately 38% of all Xpert MTB/RIF testing occurred in the Yangon region, and the proportion of individuals who were rifampicin resistant was significantly higher in this region compared to all other regions combined, 13.7% versus 7.5%. In the multivariate model (see Table 2), history of previous treatment and younger age were the largest risk factors for resistance in the study cohort which has been consistently demonstrated previously [6, 8–10, 12, 28–30]. Additionally, the increased risk for rifampicin resistance among females identified in our study was consistent with multiple other population-based studies [7, 8, 10, 28], but did conflict with smaller studies that have found male sex associated with an increased risk of resistance [13, 14]. Positive HIV status appeared to be protective in our dataset, however this is likely driven by the selection bias of Xpert MTB/RIF testing criteria during the study period where all HIV positive individuals at risk for TB were included in the testing algorithm.

Rifampicin resistance was driven primarily by the Xpert MTB/RIF probe E accounting for 75% of all resistance determinations. Probe E encompasses *rpoB* codons 528 to 533, and is likely associated with the *rpoB* 531 TTG mutation, the most common mutation associated with rifampicin resistance in this genetic region [31, 32]. This is similar to results from a recent studies in Pakistan and Ethiopia where a similar percentage of rifampicin-resistant isolates was associated with mutations on probe E [2, 33]. Less than 3% of resistance determination was caused by multiple probes, slightly higher than the previously reported multiple mutation prevalence detected by Xpert MTB/RIF of approximately 1% [2].

### Limitations

This dataset included only individuals who met Xpert MTB/RIF testing criteria and by definition were at increased risk of rifampicin resistance with the exception of individuals living with HIV, and therefore both gender and age distributions do not necessarily represent the entire cohort of individuals who were tested for TB during the study period and may not accurately represent positivity rates among individuals at risk for *Mtb* in Myanmar. In addition, there was no reference sequencing data to confirm single nucleotide polymorphisms or the accuracy of multiple mutations associated with resistance as detected by the Xpert MTB/RIF assay.

## Conclusions

Males accounted for the majority of *Mtb* positive cases in Myanmar and are at highest risk for TB disease throughout most of their adulthood, requiring continued focus on testing for this population. In addition, our analyses indicate that targeted testing among both males and females age 16–20 is warranted as our data indicate this age group has the highest proportion of *Mtb* positive results. Further evaluation is needed to identify age-specific barriers to testing that may contribute to these differential positivity rates. Moreover, among *Mtb* positive individuals both males and females less than age 30, and women overall were diagnosed with rifampicin resistance at higher rates than individuals older than age 30, or men overall. These higher risk sub-populations, males and females less than age 30, and females in all age categories, along with individuals who have been previously treated for TB, should also be targeted for Xpert MTB/RIF testing.

## Data Availability

The datasets used and/or analyzed during the current study are available from the corresponding and/or senior author on reasonable request.

## Declarations

The need for informed consent was waived and the study was approved as conducted by the Ethics Review Committee, Department of Medical Research, Ministry of Health and Sports, the Republic of the Union of Myanmar (Approval number – Ethics/DMR/2018/155). All analyses were performed in accordance with guidelines and regulations for working with de-identified data. In addition, ethics approval was sought from the University of California San Diego Institutional Review Board, La Jolla, CA, USA, however the study was determined to be exempt from review.
